# Influence of Low Magnetic Fields on Hydrogen Evolution Reaction Performance of NiCoP Electrocatalysts

**DOI:** 10.1002/cphc.202500004

**Published:** 2025-05-27

**Authors:** Vishwanath Ankalgi, Mohammed Arkham Belgami, Mihir Sahoo, Debabrata Mishra, Erdenebayar Baasanjav, Kalpataru Pradhan, Sang Mun Jeong, Chandra Sekhar Rout

**Affiliations:** ^1^ Center for Nano and Material Sciences Jain University Jain Global Campus, Jakkasandra Ramanagaram Bangalore 562112 India; ^2^ Graz University of Technology Graz 8010 Austria; ^3^ Department of Physics and Astrophysics University of Delhi Delhi 110007 India; ^4^ Theory Division Saha Institute of Nuclear Physics A CI of HBNI Kolkata 700064 India; ^5^ Department of Chemical Engineering Chungbuk National University Cheongju Chungbuk 28644 Republic of Korea

**Keywords:** electrocatalysis, hydrogen evolution reaction, magnetic fields, nickel cobalt phosphide, spin polarization

## Abstract

Developing nonprecious, high‐efficiency, and durable electrocatalysts for H_2_ evolution in acidic media is highly desirable. Extensive research has focused on improving electrocatalyst activity through techniques like defect engineering, composite formation, and doping. Here, nickel–cobalt phosphide (NiCoP) nanorods’ electrocatalytic activity enhancement by applying an external magnetic field is reported. The nanorods are synthesized via a simple hydrothermal method. When coated onto carbon paper, the NiCoP nanorods exhibit an overpotential of 100 mV at 10 mA cm^−2^. However, under a low magnetic field of 2000 G, the overpotential reduced to 62 mV at 10 mA cm^−2^ in 0.5 m H_2_SO_4_, demonstrating that the external magnetic field positively affects the kinetic process of the NiCoP nanostructure. The improved mass transport through the Lorentz force and the uniform alignment of magnetic moments of the material in the presence of a magnetic field serve for the purpose of enhanced hydrogen evolution reaction (HER) activity. The density functional theory‐based calculations support this scenario that the spin alignment can boost HER activity. These results suggest an alternative strategy for further improving the HER properties of electrocatalysts by utilizing an external magnetic field.

## Graphical abstract

Herein, electrocatalytic activity enhancement is reported of NiCoP nanorods by applying an external magnetic field. The NiCoP nanorods exhibit an overpotential of 100 mV at 10 mA cm^−2^ without magnetic field. However, under low magnetic field of 2000 G, the overpotential reduces to 62 mV at 10 mA cm^−2^ in 0.5 m H_2_SO_4_, demonstrating that external magnetic field positively affects the kinetic process of the NiCoP nanostructure.

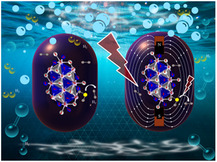

## 1 Introduction

With major developments in the energy industry, molecular hydrogen has become a more efficient and greener substitute for conventional fossil fuels.^[^ ^1^, ^2^ ^]^ A shift toward a hydrogen‐driven economy is anticipated due to hydrogen's eco‐friendly, nonpolluting combustion byproducts and superior calorific value. With the highest gravimetric energy density of 142 MJ kg^−1^ (upper heating value) among all chemical fuels, hydrogen is favored as a clean energy source.^[^ ^3^ ^]^ Although there are several ways to produce hydrogen, such as biomass electrolysis, carbonation (pyrolysis), natural gas oxidation, steam methane reforming and photo/electrochemical splitting of water, the HER, or electrochemical splitting of water, is becoming more widely acknowledged as the most promising, economically viable way to produce hydrogen.^[^ ^4^ ^]^ This method is unique in that it can produce hydrogen in both qualitative and quantitative forms, which makes it a vital component of the shift to sustainable energy sources. Given the global emphasis on transitioning toward green hydrogen production through electrocatalysis, a key factor is to investigate the possible ways to employ effective and stable electrocatalysts. Because of their better catalytic characteristics, stability, and affordability compared to precious metal‐based catalysts like platinum, bimetallic phosphides have emerged as potential options among the numerous catalysts investigated.^[^ ^5^ ^]^

Because of the mutually advantageous actions of the two metals, bimetallic phosphides have demonstrated exceptional performance in HER while providing a balance between activity, stability, and cost. Transition metal phosphides, such as nickel phosphide (Ni_2_P) and cobalt phosphide (CoP), as well as their bimetallic counterparts, have garnered significant research interest due to their exceptional catalytic characteristics.^[^ ^6^, ^7^, ^8^ ^]^ These properties originate from the interplay between the metal d‐orbitals and phosphorus p‐orbitals within the material's structure. The unique electronic configuration of transition metal phosphides enables efficient adsorption of protons and facile desorption of hydrogen at low overpotentials, rendering them highly promising catalysts for the HER with enhanced efficiency.^[^ ^9^ ^]^ Bimetallic phosphide, i.e., nickel–cobalt phosphide (NiCoP) has attracted a lot of attention because of its remarkable electrocatalytic activity in the HER.^[^ ^10^ ^]^ This material, in consideration, possesses a distinctive electronic structure that results from the combined actions of its component metals, cobalt and nickel. These two metals work together to provide a hybrid electrical environment that best supports the HER process.^[^ ^11^, ^12^ ^]^ Because of its moderate hydrogen atom binding strength, nickel is known to improve hydrogen desorption, whereas cobalt is essential for hydrogen adsorption. This interplay between nickel and cobalt allows NiCoP to achieve an optimal balance between hydrogen adsorption and desorption, resulting in improved catalytic performance for HER compared to their monometallic counterparts.^[^ ^13^ ^]^ On the other hand, phosphorus plays a crucial role in NiCoP. It not only helps to stabilize the catalyst's structure but also boosts the electron density around the metal sites. This increase in electron density is key to supporting proton reduction processes, which are critical for effective HER activity. Additionally, phosphorus enhances the overall stability of the catalyst, ensuring consistent performance throughout electrochemical reactions.^[^ ^14^ ^]^

A key challenge in electrocatalysis is reducing the overpotential and improving the electrocatalytic performance. Techniques, like defect engineering,^[^ ^15^ ^]^ structural engineering, doping, and phase engineering, have been explored to the bottleneck stage.^[^ ^16^, ^17^ ^]^ However, these strategies are often complex and limited. Beyond improving the inherent activity of catalysts and increasing their loading mass, new intensification strategies involving external excitations such as magnetic fields,^[^ ^18^, ^19^, ^20^ ^]^ light,^[^ ^21^, ^22^, ^23^, ^24^, ^25^ ^]^ electric fields,^[^ ^26^, ^27^, ^28^, ^29^ ^]^ and stress^[^ ^30^, ^31^ ^]^ have become a trend in electrochemistry. These methods effectively improve mass transfer on the electrode surface and alter reaction kinetics. Recently, the influence of magnetic fields on electrocatalytic activity has been explored in several studies, particularly for reactions involving transition metals with intrinsic magnetic properties. The reported studies have briefly examined the influence of magnetic fields on enhancing electrocatalytic activity from different perspectives. For instance, to study the effect of spin polarization, Ren et al. have studied the spin polarized kinetics of oxygen evolution reaction, in which the group has investigated the effect of magnetic field to polarize the magnetic moments of the ferromagnetic (FM) electrocatalyst which in‐turn has a pronounced effect in decreasing the Tafel slope value from 109 to 87.8 mV dec^−1^.^[^ ^32^ ^]^ These magnetic fields can also directly improve electrocatalytic performance by increasing electron transfer efficiency or regulating the reaction pathway by influencing the electrocatalyst's spin arrangement.^[^ ^33^, ^34^ ^]^ Additionally, magnetic fields can indirectly affect water electrolysis, such as increasing the local temperature of electrocatalysts through the magnetic heating effect of AC magnetic fields or improving diffusion via the Magnetohydrodynamic (MHD) effect.^[^ ^18^, ^35^ ^]^ Huang et al. reported the magnetic heating of electrocatalysts by means of Neel's relaxation, as the case the NiSe_2‐x_ electrocatalysts’ intrinsic catalytic activity was increased in the presence of external alternating magnetic field, thereby resulting in enhanced HER performance.^[^ ^36^ ^]^ To investigate the MHD effect, Hegde et al. reported a significant improvement in the HER efficiency of Ni–W alloys under a magnetic field, the group concludes by stating the pronounced MHD‐induced convection of hydrogen bubbles liberation from the surface of the electrode.^[^ ^37^ ^]^

In this work, we have hydrothermally synthesized NiCoP having nanorods‐like morphology. The HER behavior of the material was analyzed in acidic medium by varying the external magnetic field strength from 0 to 2000 G. Interestingly we have observed a decrease in overpotential from 100 to 64 mV at 10 mA cm^−2^ current density.

Applying an external magnetic field has significantly affected the catalytic properties of materials, especially those with magnetic elements like NiCoP, for instance. Ni and Co, which have unpaired d‐electrons, generate magnetic moments, which align in response to a magnetic field. This alignment, called magnetic moment alignment, can alter the material's electronic structure, resulting in changes to its surface energy and catalytic performance. Density functional theory (DFT) calculations were carried out as support for the experimental findings. Our DFT investigations demonstrate that the HER performance is improved when the spin moments of the Co atoms are aligned parallel to each other. In this case, the Co site in NiCoP, the primary site for hydrogen adsorption, shows the optimal binding energy to the atomic hydrogen. A decrease in the Gibbs free energy for hydrogen adsorption (ΔG_H*_), a key indicator of HER activity, results from this optimized‐binding‐energy configuration. This approach offers a new strategy for enhancing the performance of nonprecious metal catalysts in water splitting, especially for sustainable hydrogen production technologies. Future studies will likely focus on refining the strength and direction of the magnetic field to optimize catalytic benefits and investigating other transition metal‐based systems where magnetic field‐induced effects can boost electrocatalytic activity.

## 2 Results and Discussions

### 2.1 Synthesis and Structural Characterizations

**Figure 1:**
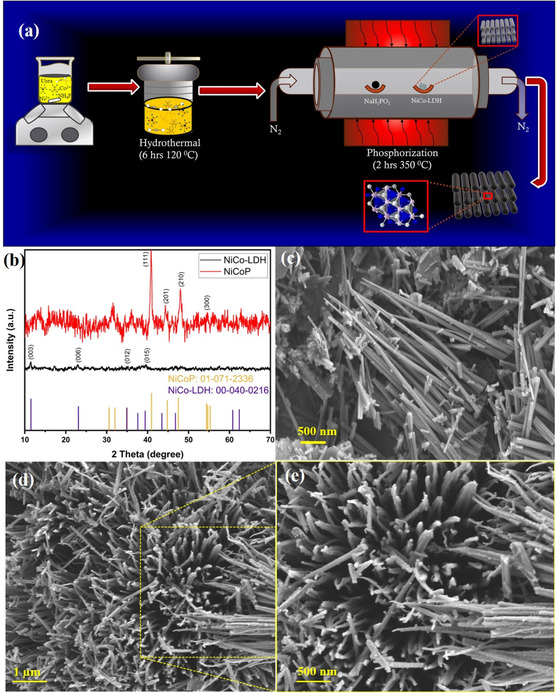
a) Schematical representation of the synthesis procedure, b) XRD pattern of NiCoP, and c–e) scanning electron microscope images of NiCoP with different resolutions.

The NiCoP nanorods were obtained as a result of simple hydrothermal method as shown in **Figure** **1**a, wherein the precursors of Ni and Co were stirred in deionized  water containing urea and ammonium fluoride. The finely dissolved mixture was then heated in an autoclave for six hours at 120 °C, resulting in the formation of NiCo‐layered double hydroxide (NiCo‐LDH), a part of which was subjected to preliminary characterizations for confirmation. The conversion of NiCo‐LDH to NiCoP proceeds via a gas‐phase phosphorization process, typically involving thermal treatment in the presence of a phosphorus source. During this process, the reductive environment in the closed medium allows the hydroxide anions (OH^−^) in NiCo‐LDH to be gradually replaced by phosphorus atoms, forming metal–phosphorus bonds (M—P, where M = Ni, Co) and leading to the development of a crystalline NiCoP phase.^[^ ^38^ ^]^ As indicated in Figure 1b, the X‐ray diffraction (XRD) pattern of the precursor NiCo‐LDH shows characteristic peaks at 2θ values of 11.3°, 22.8°, 35.1°, and 39.7°, corresponding to the (003), (006), (012), and (015) planes of the layered double hydroxide structure (ICDD: 00‐040‐0216). After phosphorization, these peaks disappear and are replaced by distinct reflections at 40.86°, 44.4°, 47.8°, 54.4°, 54.7°, and 55.8°, which are indexed to the (111), (201), (210), (300), (002), and (211) planes of the hexagonal NiCoP phase (ICDD: 01‐071‐2336).^[^ ^39^ ^]^ This shift in the diffraction pattern confirms the successful conversion of the hydroxide phase into the phosphide phase. The shift of the peaks to higher angles reflects lattice contraction due to P^3−^ incorporation. The (111) reflection shows the highest intensity among the observed peaks, consistent with the standard diffraction pattern for NiCoP (ICDD 01‐071‐2336). This suggests that the (111) facet is among the more dominant planes exposed, which could influence the surface reactivity relevant to HER.^[^ ^40^ ^]^

As shown in the field‐emission scanning electron microscope (FESEM) images (Figure 1c–e), the NiCo‐LDH precursor exhibits well‐defined nanorod morphology. After phosphorization, the NiCoP retains the nanorod architecture, as evident in the high‐resolution images. However, a closer inspection reveals subtle surface roughening and a slightly more porous structure, which is likely due to partial etching and gas evolution during the thermal phosphorization.^[^ ^41^ ^]^ This observation indicates that the transformation process occurs topotactically, preserving the overall morphology while modifying the crystal phase and microstructure. Using energy‐dispersive X‐ray analysis, elemental mapping of NiCoP was carried out, confirming the homogeneous distribution of Ni, Co, and P in the sample (Figure S1, Supporting Information). According to the quantitative study, the elements’ atomic percentages were 38.5, 17, and 44.5 of Co, Ni, and P, respectively. This regular distribution of components within the NiCoP nanorods guarantees the material's compositional homogeneity and confirms the material's successful synthesis, which is crucial for its catalytic performance. The increased atomic percent of Co compared to Ni is attributed to enhanced HER by virtue of enhancing electron transfer capabilities and stabilizing active sites.^[^ ^42^ ^]^

**Figure 2:**
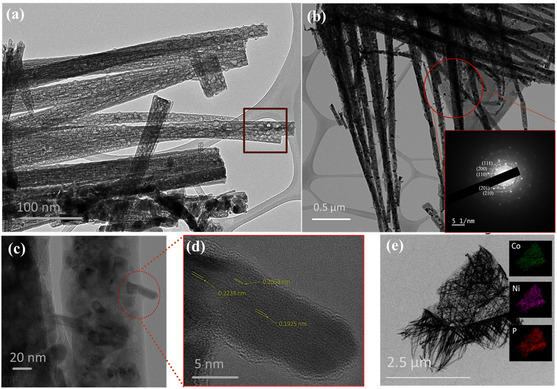
a) TEM images, b) with inserted selected area electron diffraction (SAED) pattern, and c,d) HRTEM image, and e) element mapping of NiCoP.

**Figure 3:**
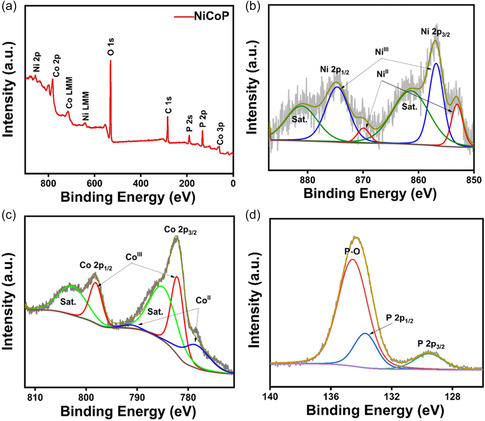
a) XPS survey spectrum of NiCoP, XPS spectra of b) Ni 2*p*, c) Co 2*p*, and d) P 2*p*.

Further, the high‐resolution transmission electron microscopy (HRTEM) was carried out. **Figure** **2**a–b highlights the nanorod‐like morphology of NiCoP; moreover, Figure 2a exhibits many pores distributed along the nanorods after the phosphorization. The formation of pores observed in the nanorods can be attributed to the phosphorization process, where the replacement of hydroxyl groups in NiCo‐LDH by phosphorus species leads to lattice shrinkage and mass loss, resulting in pore generation. Additionally, during this transformation, smaller crystallites may dissolve and recrystallize, leaving behind voids in the structure. This phenomenon aligns with well‐established mechanisms such as Ostwald ripening and the Kirkendall effect, both of which have been widely reported in literature to explain the evolution of porosity and hollow structures during nanomaterial synthesis.^[^ ^43^ ^]^ The presence of these pores can favor in increasing the surface area, stabilizing active sites, and promoting synergistic effects between Ni and Co.^[^ ^42^, ^44^ ^]^ The inset of Figure 2b confirms the crystalline nature of NiCoP with prominent diffraction spots corresponding to (111), (200), (110), (201), and (210) crystal planes. To further validate the XRD results, Figure 2c–d illustrates interplanar distances of 0.220, 0.201, and 0.190 nm, which correspond well with the d‐spacing of the (111), (201), and (210) planes of NiCoP. These findings confirm the crystallographic alignment and structural integrity of the synthesized NiCoP nanorods. The energy‐dispersive X‐ray spectroscopy in Figure 2e further validates the homogeneous distribution of Co, Ni, and P elements in the NiCoP nanorods. The elemental composition and oxidation states of NiCoP were characterized using X‐ray photoelectron spectroscopy (XPS). The survey spectrum (**Figure** **3**a) confirms the presence of Ni, Co, and P. The Ni 2*p* spectrum (Figure 3b) shows peaks at 853.5 and 870.5 eV corresponding to Ni^2+^ 2*p*_3/__2_ and Ni^2+^ 2*p*_1/__2_, respectively, along with peaks at 856.3 and 874.0 eV attributable to Ni^3+^ states. Shake‐up satellite peaks at 861.3 and 879.4 eV further confirm the presence of oxidized nickel species. The Co 2*p* spectrum (Figure 3c) displays peaks at 778.67 and 782.1 eV for Co^2+^ and Co^3+^ 2*p*_3/__2_, and at 792.1 and 798.0 eV for Co^2+^ and Co^3+^ 2*p*_1/__2_, respectively, along with satellite peaks at 784.8 and 802.7 eV. These results confirm the coexistence of mixed valence states (Co^2+^/Co^3+^ and Ni^2+^/Ni^3+^), which are known to improve electron transport and facilitate redox reactions at the electrode–electrolyte interface, thus enhancing catalytic activity.^[^ ^40^, ^45^ ^]^ The P 2*p* spectrum (Figure 3d) exhibits peaks at 129.5 and 130.4 eV, confirming the formation of metal–phosphide bonds (P—M), while the peak at 133.6 eV corresponds to oxidized phosphorus species (P—O/PO_x_), likely due to surface oxidation.^[^ ^46^, ^47^ ^]^

### 2.2 Electrocatalytic and Magnetic Studies

**Figure 4:**
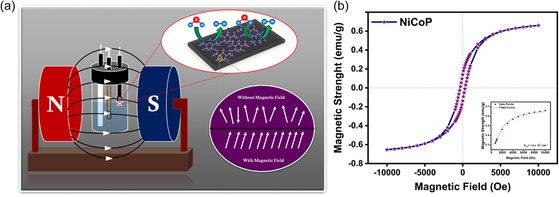
a) Schematic representation of electrochemical measurement setup in the presence of an external magnetic field, and b) M–H curve of NiCoP.

To carry out the electrochemical measurements in the presence of an external magnetic field, an external electromagnet with variable magnetic strength was used (**Figure** **4**a). Prior to electrochemical analysis, to study the magnetic characteristics of NiCoP, a vibrating sample magnetometer (VSM) was employed. Figure 4b represents the M–H curve of NiCoP; the saturation magnetic field and saturation magnetization were calculated to be 7 kOe and 0.71 emu g^−1^, respectively, and the coercivity value of 329 Oe further corroborates the soft FM nature of NiCoP nanorods. Inset of Figure 4b represents the fitted M–H curve of NiCoP using the law of approach to FM saturation given by Equation (1).^[^ ^48^ ^]^

(1) $$M=M_{S}\left(1-\frac{a_{1}}{H}-\frac{a_{2}}{H^{2}}\right)+\chi_{P}H$$

where *χ*_P_ is the high field susceptibility, *M*_S_ is the saturation magnetization, and *a*_1_ and *a*_2_ are constants. The anisotropy constant was also calculated using the following Equation (2) and was found to be *K*_eff_ = 1.6 × 10^3^ Jm^−3^.^[^ ^48^ ^]^ This low value of *K*_eff_ suggests that the NiCoP exhibits an easy magnetization reversal.
(2)

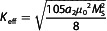




The HER performance of NiP, CoP, Pt/C, and NiCoP coated onto a carbon paper substrate was initially evaluated in 0.5 m H_2_SO_4_. The linear sweep voltammetry (LSV) curves for these materials are shown in (Figure S2a, Supporting Information). The data clearly demonstrate that NiCoP exhibits significantly enhanced HER activity compared to NiP and CoP individually, indicating a synergistic effect in the bimetallic phosphide, and the activity of NiCoP approached the performance of Pt/C. The Tafel slope values recorded for NiP, CoP, and NiCoP were 183, 172, and 170 mV dec^−1^, respectively (Figure S2b, Supporting Information). The theoretical Tafel slopes for the Volmer, Heyrovsky, and Tafel steps are 120, 41, and 30 mV dec^−1^, respectively, which suggests that our material follows the Volmer–Heyrovsky reaction mechanism, where the Volmer step (adsorption of hydrogen) is the rate‐determining step in the electrochemical process. This indicates that NiCoP not only lowers the overpotential for HER but also operates through an efficient proton adsorption and electron transfer mechanism, making it a promising catalyst for hydrogen evolution.

**Figure 5:**
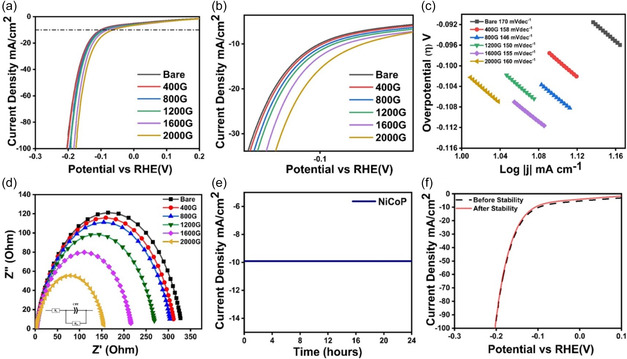
a–b) LSV curves for NiCoP in 0.5 m H_2_SO_4_, c) Tafel plot, d) EIS plot of NiCoP at different magnetic field strengths from 0 to 2000 G, e) pontentiostatic CA curves at static potential for 24 h, and f) LSV curves comparing the HER activity of NiCoP before and after stability.

The strength of the external electromagnet was varied from 0 to 2000 G to study the effect of the external magnetic field on the HER performance of the NiCoP electrocatalyst. The LSV curves and Tafel plots of the sample under the effect of the magnetic field are shown in **Figure** **5**a–c. The overpotential at 10 mA cm^−2^ and Tafel slope values found a substantial decrease from 100 to 62 mV and 170 to 146 mV dec^−1^, respectively, when the magnetic field strength varied from 0 to 2000 G. The corresponding electrochemical impedance spectroscopy (EIS) spectra of the material at different magnetic field strength are depicted in Figure 5d, which highlights the reduction in charge transfer resistance. Further to evaluate the stability of the electrocatalyst, it was subjected to time‐dependent chronoamperometric (CA) current performance at 100 mV static potential as depicted in Figure 5e. The LSV curves before and after stability (Figure 5f) display maximum retention in performance in terms of overpotential at 10 mA cm^−2^.

The appreciable decrease in the overpotential and Tafel slope values can be attributed to the generation of Lorentz force and spin polarization of the electrocatalyst in the presence of magnetic field. The Lorentz force which is given by Equation (3) is generated in the system in the presence of a magnetic field, here *F* is the Lorentz force, *q* is the charge, *v* is the velocity of the charge, *B* is the magnetic induction intensity, and *E* is the electric field strength generated depending on the strength of *B*.^[^ ^49^ ^]^

(3) F=q(E+v×B)

This force tends to promote mass transfer onto the electrode surface, and thereby reduce the charge transfer resistance, the gradual reduction in the charge transfer resistance can be visualized in Figure 5d, where the increase in the magnetic field from 0 to 2000 G decreases the size of semicircle in the EIS plots which suggest the change in *R*_ct_ value of the electrocatalyst with the increase in the applied magnetic field strength. The Lorentz force also promotes the release of gas bubbles from the electrode surface, which improves the availability of active sites for further reaction. This is done by virtue of the Lorentz force influencing fluid motion in the electrolyte, which leads to generation of convection current in the medium. These currents affect the motion of electrolyte material, the ions which are subjected to continuous Lorentz force normal to their direction of movement migrate in a circular path. This kind of motion reduces the thickness of the diffusion layer near the electrode surface by causing rapid release of bubbles from the surface, as a result of which the movement of reactants toward the electrode is increased.^[^ ^49^ ^]^

**Figure 6:**
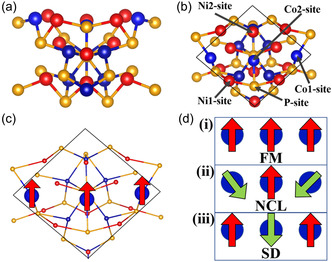
a) side and b) top views of NiCoP (111) surface with different adsorption sites for H atom. c) Schematic view of FM surface with spin orientation of Co atoms at the top layer. d) Three different types of spin configuration surfaces: 1) FM, 2) NCL, and 3) SD surfaces.

Further, to verify the underlying mechanism for the enhancement in HER activity under the influence of an external magnetic field, we performed DFT studies. As shown in **Figure** **6**a, the NiCoP (111) surface with a 1 × 1 supercell size along the ab direction and three atomic layers of thickness ≈6.1 Å along the *c* direction was taken into consideration for the study of HER activity. Each layer is composed of three atoms of Ni, Co, and P, respectively. Five inequivalent sites on the top of the NiCoP (111) surface can be taken into consideration for hydrogen adsorptions [see Figure 6b] and are designated as Co1, Co2, Ni1, Ni2, and P sites. Based on an initial collinear magnetic calculations, the ground state of the system is FM, where each of the three cobalt atoms in the top layer is aligned ferromagnetically and has a significant magnetic moment of 1.2 μ_B_. However, the magnetic moments of Ni and other cobalt atoms are very small. Then, we proceed to analyze the noncollinear (NCL) magnetic calculations to reveal the impact of the magnetic field on the surface catalytic activity, with particular attention paid to the noncollinearity of the magnetic moments of the three cobalt atoms present at the top layer. First, a surface, known as the FM surface, is created by fixing all of the cobalt atoms’ magnetic moments parallel to the supercell's *c*‐axis. This gives rise to a total magnetic moment of 4.54 μ_B_. The spin orientations of the cobalt atoms of the FM surface are schematically shown in Figure 6c. We considered two other surfaces where the spin of cobalt atoms is oriented differently for further studies. For instance, we have maintained the magnetic moments of the three cobalt atoms at an angle of 120°, 0°, and 240° with respect to the supercell's *c*‐axis, respectively, and named the system as NCL surface (Figure 6d). The nature of this system is more toward an anti‐FM spin arrangement, since all of the magnetic moments of Co atoms, both in‐ and out‐of‐plane, are zero. However, the net magnetic moment of the top‐layer Ni atoms is 0.33 μ_B_. Similarly, we have considered another system where spins of cobalt atoms are oriented at 0°, 180°, and 0° with respect to the *c*‐axis of the supercell and termed it as spin‐disorder (SD) surface. The NCL and SD surfaces have energies 13.3 and 2.5 meV higher than that of the FM surface, respectively. So, it is highly likely that the spins of Co atoms in experiments are randomly oriented, and the application of a magnetic field orients them in one direction.

**Figure 7:**
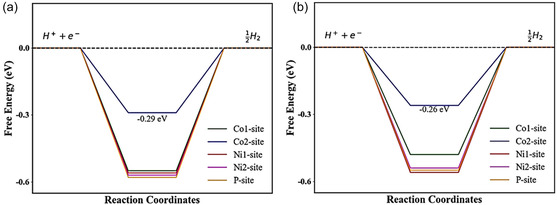
Gibbs free energy diagram for a) NCL and b) FM surfaces.

Next, to characterize HER activities, Δ*G*_H*_ at various H adsorption sites is computed using the above three surfaces. However, Δ*G*_H*_ values at different sites for both NCL and SD surfaces are very much similar to each other. Hence, we have compared the free energy profiles for the NCL surface with the FM surface to understand the effect of magnetic field on HER catalysis (as shown in **Figure** **7**a–b. A low Δ*G*_H*_ value of −0.29 eV is found when the H atom is adsorbed to the Co2 sites of the NCL surface. This indicates that the Co2 site is the catalytic active site. However, in the FM surface, Δ*G*_H*_ is equal to −0.26 eV, suggesting better catalytic activity in comparison to the NCL surface. This observation is in good agreement with the experimental results, where the magnetic field enhances the catalytic activity toward HER on the NiCoP surface. Furthermore, for the Co1, Ni1, Ni2, and P‐sites of the FM (NCL) surface, the Δ*G*_H*_ values are –0.48 (−0.55), –0.56 (−0.58), –0.54 (−0.58), and –0.54 (−0.58 eV), respectively. Despite being catalytically inert sites on both surfaces, the FM surface has lower absolute values of Δ*G*_H*_ than the NCL surface for these four sites.

**Figure 8:**
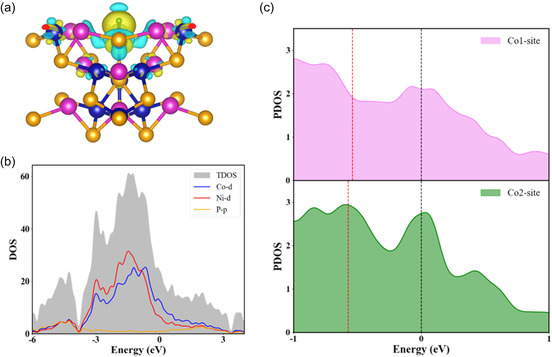
a) Side view of charge density difference plot when H is adsorbed at Co2‐site of NiCoP (111) surface. b) Total and projected density of states of NiCoP (111) surface. c) Partial density of states at Co1 and Co2 sites along with d‐band centers (dashed red lines). Fermi level is set at 0 eV.

Next, we compute the charge density difference at the interface when H is adsorbed at the Co2‐site in order to visualize the charge transfer, as shown in **Figure** **8**a. It is clear that a charge depletion area is located close to the Co2‐site, whereas the electrons are concentrated around the H atom. From the Bader charge calculations,^[^ ^50^ ^]^ when a H atom is adsorbed on top of a Co atom, it gains 0.26 e of charge, but 0.28 e of charge is gained by H for adsorption at the Ni‐site. In addition, the projected density of states of the NiCoP(111) surface is plotted in Figure 8b. Strong hybridization between 3*p* orbitals of P sites and the 3*d* orbitals of both Ni and Co is observed above the Fermi level, whereas interaction between the 3*d* states of Ni and Co atoms is seen below it. This strong interaction between the orbitals of the constituent atoms of the substrate is likely the reason behind the strong binding of the hydrogen atom at different sites. On the other hand, by computing the d‐band centers of Co atoms, we have attempted to examine the variations of Δ*G*_H*_ by calculating the strength of chemical interactions between H and the substrates at different Co sites, which can be calculated as follows (Equation (4)).^[^ ^51^ ^]^

(4) $$\Delta E\propto -\frac{V^{2}}{\varepsilon_{d}-\varepsilon_{s}}$$

where *V* stands for the coupling matrix element, *a* constant. The relative position of the *d*‐band center with respect to the Fermi energy is denoted by *ε*_d_, while the energy level of the adsorbate state is represented by *ε*_s_, which is fixed at 0 eV. The d‐band center, *ε*_d,_ can be calculated from the following Equation (5).

(5) $$\varepsilon_{d}=\frac{\int_{0}^{\infty }x\rho \left(x\right)dx}{\int_{0}^{\infty }\rho \left(x\right)dx}$$

where, the values of energy (abscissa) and projected d‐orbital density of states of Co atoms (ordinate) are represented by *x* and *ρ*(*x*), respectively (Figure 8c). Chemical bond energy is primarily described by states in the −1–0 eV energy range.^[^ ^52^, ^53^ ^]^ In contrast to the Co2‐site, the d‐band center for the Co1‐site is located near the Fermi level. As a result, H binds to the Co1‐site more firmly, making H_2_ desorption challenging. The reaction intermediate must moderately bind to the substrate for a good catalyst. Because the chemical bond between Co and H is optimum in Co2‐site adsorption, the HER activity is more prominent at the Co2 site.

### 2.3 Postanalysis Characterization

To evaluate the structural and morphological stability of the electrode material after long‐term cycling, postcharacterization analysis were conducted using XRD and FESEM (Figure S3a–c, Supporting Information). The XRD pattern of the cycled electrode retains the main diffraction peaks observed in the fresh sample, albeit with reduced intensity, indicating partial structural preservation. A new diffraction peak around 42.8° was observed, which can be attributed to the formation of Ni_3_P and Co_2_P phases, corresponding to the (330) plane of Ni_3_P (JCPDS No. 00‐034‐0501) and the (211) plane of Co_2_P (JCPDS No. 00‐032‐0306), suggesting partial oxidation during electrochemical cycling. FESEM images reveal that the rod‐like morphology is largely preserved, though noticeable agglomeration of nanorods and adherence of electrolyte residues were observed. These results confirm the material's structural integrity and compatibility with prolonged electrochemical operation.

## 3 Conclusions

In summary, we propose an alternate approach to boost the NiCoP electrocatalyst's HER activity by applying an external magnetic field. The introduction of the magnetic field alters the local electron distribution, thereby enhancing the HER performance of the NiCoP electrocatalyst. Furthermore, this impact alters the direction of electron migration, which promotes electron localization at the active sites. Additionally, the orientation of magnetic moments of the electrocatalyst material in the presence of a magnetic field tends to decrease the free energy of the reaction and significantly boosts catalytic activity. As a result, the Tafel slope drops from 170 to 146 mV dec^−1^, and the overpotential needed for the NiCoP electrocatalyst drops from 100 to 62 mV at a current density of 10 mA cm^−2^. Comparing the HER process with and without the magnetic field confirms that the magnetic field plays a key role in enhancing the catalytic activity. These techniques of enhancing the activity can still be clearly understood and further developed to pave the way for exceptional research and groundbreaking insights.

## 4 Experimental Section

4.1

4.1.1

##### Synthesis of NiCoP

The Ni–Co LDH nanorods were first synthesized via a hydrothermal method. In a typical procedure, 0.291 g (1 mmol) of Co(NO_3_)_2_·6 H_2_O and 0.5 mmol of Ni(NO_3_)_2_·6 H_2_O were dissolved in 28 mL of deionized water containing 3 mmol of urea and 1 mmol of NH_4_F. The mixture was stirred at room temperature for 20 min to obtain a homogeneous pink solution, which was subsequently transferred into a Teflon‐lined stainless steel autoclave. The autoclave was sealed and heated at 120 °C for 6 h. After naturally cooling to room temperature, the resulting precipitate was collected and thoroughly washed with deionized water and ethanol several times. The cleaned product was then dried overnight at 60 °C to obtain the Ni–Co LDH nanorods. For the phosphorization step, NaH_2_PO_2_·H_2_O was employed as the phosphorus source. The dried Ni–Co LDH nanorods and NaH_2_PO_2_·H_2_O (in a mass ratio of 1:10) were separately placed at the downstream and upstream ends of an alumina boat, respectively. The boat was then placed at the center of a tube furnace, which was purged with nitrogen gas. The temperature was ramped to 350 °C at a rate of 2 °C min^−1^ and maintained for 2 h. After naturally cooling to room temperature under nitrogen, the final NiCoP nanorods were collected for further characterization.^[^ ^46^ ^]^

##### Physical and Chemical Characterizations

XRD using CuKα radiation with a wavelength of ≈0.154 nm was employed to assess the phase purity and crystallinity of the material within the 2θ range of 10°–80°, at a scan rate of 1° min^−1^ (Dmax/1200, Rigaku Company, Japan). The internal structure, surface morphology, and elemental composition were analyzed using FE‐SEM (Carl Zeiss, Ultra plus, LEO‐1530), transmission electron microscopy (TEM; JEM‐2100F, UHR, JEOL, KBSI), and energy‐dispersive X‐ray spectroscopy. XPS (PHI Quantera‐II, Al Kα radiation, Ulvac‐PHI) was utilized to investigate the valence states of the elements and near‐surface elemental compositions. The material's magnetic properties were measured using a Microsense Model ADE EV9 VSM. The electrocatalyst was electrochemically evaluated to ascertain its physicochemical parameters using the Corrtest electrochemical workstation (CS350) and related CS Studio software.

##### Electrochemical Methods

The HER activity of the NiCoP electrocatalyst was measured in acidic electrolyte (0.5 m H_2_SO_4_), by traditional three electrode cell system. The LSV was recorded at a scan rate of 5 mV s^−1^. All the reported potentials were converted to reversible hydrogen electrode (RHE) using Equation (6).

(6) $$E_{\text{RHE}}=E_{\text{Ag/AgCl}}+0.059\times pH+0.197$$

where *E*_Ag/AgCl_ is the obtained potential relative to the Ag/AgCl reference electrode, *E*_RHE_ represents the potential of the reversible hydrogen electrode, and 0.197 V is the standard electrode potential for the Ag/AgCl at ambient temperature. The Tafel slope was determined using Equation (7), where *η* is the overpotential, *b* is the Tafel slope, *j* is the current density, and *a* is the constant.

(7) η=b×log(j)+a

##### Computational Method

Using the Vienna Ab initio Simulation Package (VASP) code,^[^ ^54^, ^55^ ^]^ first principles DFT were applied to investigate the structural, electronic, and catalytic aspects of NiCoP (111) surface. The Perdew–Burke–Ernzerhof version of the generalized gradient approximation was used with the projector augmented wave pseudopotential to describe the exchange‐correlation functional. For the plane‐wave basis set expansion,^[^ ^56^ ^]^ a kinetic energy cut‐off of 500 eV was considered, and using the Monkhorst‐Pack method,^[^ ^57^ ^]^ a K‐point grid of 4 × 4 × 1 was chosen to sample the first Brillouin zone. We create a vacuum of 20 Å along the *c*‐axis of the supercell to elude the interaction between the periodic images. In this setup, the system was fully relaxed until the Hellman–Feynman force on each atom is less than 0.001 eV Å^−1^, and the energy difference between the consecutive self‐consistent electronic calculations was smaller than 10^−4^ eV. To reveal the effect of external magnetic field on catalytic activity toward HER, we carried out NCL magnetic calculations with the addition of the spin–orbit coupling effect.

To predict the catalytic activity of NiCoP (111) surface, change in Gibbs free energy for hydrogen adsorption (Δ*G*_H*_), a key descriptor for HER can be calculated through computational hydrogen electrode model^[^ ^58^ ^]^ as per the following Equation (8).

(8) $$\Delta G_{H}=\Delta E_{H}+\Delta E_{\text{ZPE}}-T\Delta S_{H}$$

where Δ*E*_ZPE_ and Δ*S*_H*_ represent the difference in energy and difference in entropy between adsorbed hydrogen (H*) and H_2_ molecule (gas phase), respectively. Negligible contributions to these terms come from the catalytic surface, and hence can be neglected. In addition, adsorbed H has a very minimal vibrational entropy, and thus, almost has no effect on the term Δ*G*_H_*. At *T* = 300 K, the values of *T*Δ*S*_H*_ and Δ*E*_ZPE_ (for H*) are found to be –0.2 and 0.04 eV, respectively.^[^ ^59^, ^60^ ^]^ Hence, for HER, Δ*G*_H*_ can be determined by Δ*G*_H*_ = Δ*E*_H_ + 0.24 eV. The Δ*G*_H*_ should be small for a good catalyst. Here, the value of Δ*E*_H_ indicates that the binding energy of H with the catalytic substrate and can be evaluated from the energies of substrates (*E*_S_), substrates with H (*E*_S + H_), and molecular hydrogen ($E_{H_{2}}$) as following Equation (9).

(9) $$\Delta E_{H}=E_{\text{S + H}}-E_{S}-\frac{1}{2}E_{H_{2}}$$

## Conflict of Interest

The authors declare no conflict of interests.

## Author Contributions

**Vishwanath Ankalgi**: data curation (equal); formal analysis: (equal); investigation: (equal); methodology: (equal). **Mohammed Arkham Belgami**: data curation: (equal); formal analysis: (equal); investigation: (equal); methodology: (equal). **Mihir Sahoo**: investigation: (supporting). **Debabrata Mishra**: investigation: (supporting); software: (supporting). **Erdenebayar Baasanjav**: investigation: (supporting). **Kalpataru Pradhan**: software: (lead); supervision: (lead). **Sang Mun Jeong**: funding acquisition: (lead); investigation: (equal); writing—review & editing: (lead). **Mohammed Arkham Belgami** and **Vishwanath Ankalgi** have contributed equally to the manuscript.

## Supporting information

Supplementary Material

## Data Availability

The data that support the findings of this study are available from the corresponding author upon reasonable request.
