# Supplementary material for: Influence of Low Magnetic Fields on Hydrogen Evolution Reaction Performance of NiCoP Electrocatalysts
Source: Chemphyschem. 2025 May 27;26(14):e202500004. doi: 10.1002/cphc.202500004 (PMC12276036; doi:10.1002/cphc.202500004)
Supplement: Supplementary file 1 — Supplementary Material [file CPHC-26-e202500004-s001.pdf]

Supplementary Information

Influence of Low Magnetic Field on Hydrogen Evolution Reaction Performance of NiCoP Nanorods

Vishwanath Ankalgi, Mohammed Arkham Belgami, Mihir Sahoo, Debabrata Mishra, Erdenebayar Baasanjav, Kalpataru Pradhan, Sang Mun Jeong, Chandra Sekhar Rout**

Vishwanath Ankalgi, Mohammed Arkham Belgami, Prof. Chandra Sekhar Rout
Centre for Nano and Material Sciences, Jain University, Jain Global Campus, Jakkasandra, Ramanagaram, Bangalore-562112, India.
E-mail: csrout@gmail.com, r.chandrasekhar@jainuniversity.ac.in (CSR)

Mihir Sahoo
Graz University of Technology, Graz,8010, Austria

Dr. Debabrata Mishra
Department of Physics and Astrophysics, University of Delhi, Delhi-110007

Dr. Kalpataru Pradhan
Theory Division, Saha Institute of Nuclear Physics, A CI of HBNI, Kolkata 700064, India

Dr. Erdenebayar Baasanjav, Prof. Sang Mun Jeong, Prof. Chandra Sekhar Rout
Department of Chemical Engineering, Chungbuk National University, Cheongju, Chungbuk 28644, Republic of Korea.
E-mail: smjeong@chungbuk.ac.kr (SMJ)

MAB and VA have contributed equally to the manuscript

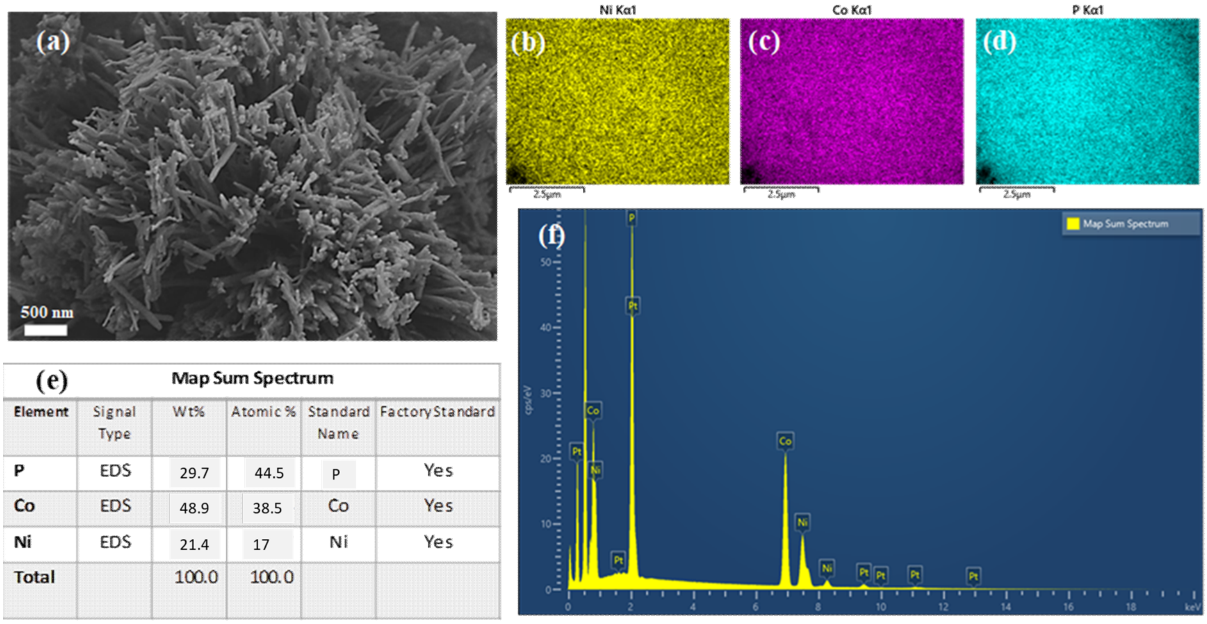

Element	Signal Type	Wt%	Atomic %	Standard Name	Factory Standard
P	EDS	29.7	44.5	P	Yes
Co	EDS	48.9	38.5	Co	Yes
Ni	EDS	21.4	17	Ni	Yes
Total		100.0	100.0		

Figure S1: (a) FESEM image showing the nanorod-like structure of the Ni-Co-P. (b), (c), & (d) E-DAX characterization images showing the atomic distribution of the Ni, Co, & P respectively. (e) & (f) depicting the map sum spectrum of the Ni-Co-P nanorods.

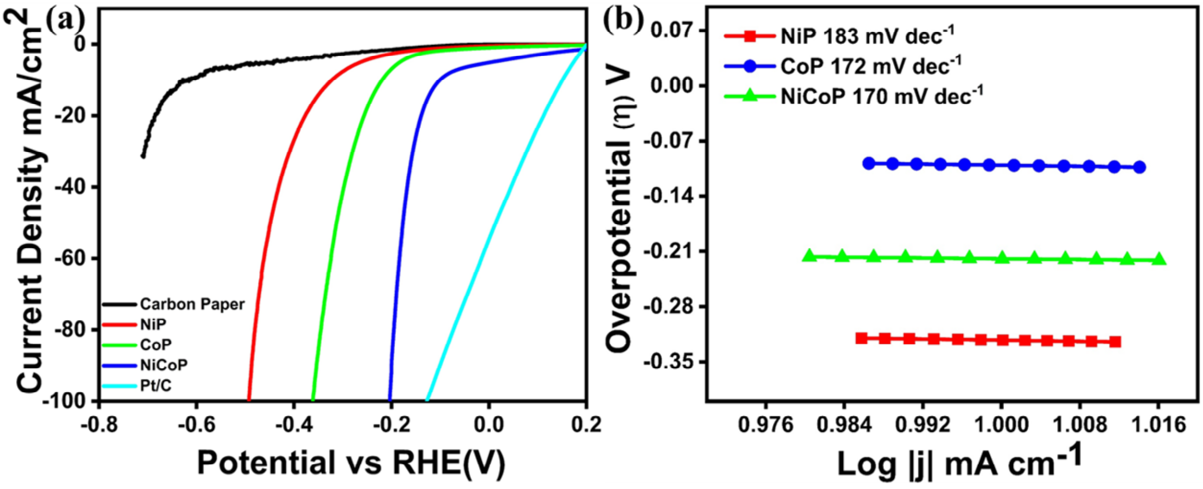

Figure S2: (a) LSV plots of the Carbon Paper, NiP, CoP, Pt/C, and NiCoP. (b) Tafel slope for the NiP, CoP, and NiCoP.

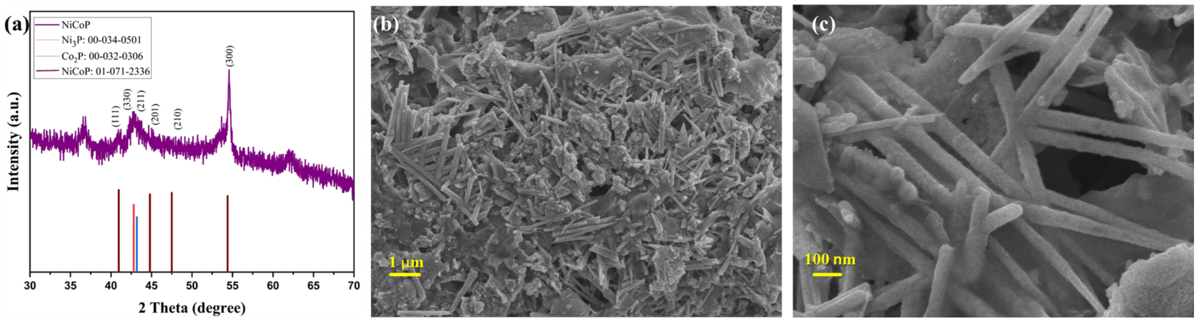

Figure S3: (a) post analysis XRD of the sample, (b) and (c) post analysis FESEM images of the sample
